# Mass chemotherapy with niclosamide for the control of *Taenia solium*: population-based safety profile and treatment effectiveness

**DOI:** 10.1016/j.lana.2024.100876

**Published:** 2024-08-30

**Authors:** Melissa T. Wardle, Samantha E. Allen, Ricardo Gamboa, Percy Vilchez, Seth E. O'Neal, Claudio Muro, Andrés G. Lescano, Luz M. Moyano, Guillermo E. Gonzalvez, Armando E. González, Robert H. Gilman, Héctor H. García, Cysticercosis Working Group in Peru (CWGP), Manuela R. Verastegui, Javier A. Bustos, Mirko Zimic, Isidro Gonzales, Herbert Saavedra, Sofia S. Sanchez, Manuel Martinez, Yesenia Castillo, Luz Toribio, Gianfranco Arroyo, Miguel A. Orrego, Nancy Chile, Holger Mayta, Monica Pajuelo, Saul Santivañez, Eloy Gonzalez-Gustavson, Luis Gomez-Puerta, Cesar M. Gavidia, Ana Vargas-Calla, Maria T. Lopez, Theodore E. Nash, Sukwan Handali, John Noh, Jon Friedland

**Affiliations:** a School of Public Health, Oregon Health & Science University and Portland State University, Portland, OR, USA; b Department of Neurology, School of Medicine, University of California, Davis, Sacramento, CA, USA; c Centro de Salud Global, Tumbes, Universidad Peruana Cayetano Heredia, Lima and Tumbes, Perú; d Facultad de Salud Pública y Administración, Universidad Peruana Cayetano Heredia, Lima, Perú; e School of Human Medicine, Universidad Nacional de Tumbes, Tumbes, Perú; f Enfermedades Transmisibles y Análisis de Salud, Organización Panamericana de la Salud OPS/OMS, Bogotá, Colombia; g School of Veterinary Medicine, Universidad Nacional Mayor de San Marcos, Lima, Perú; h Department of International Health, Bloomberg School of Public Health, Johns Hopkins University, Baltimore, MD, USA; i Instituto Nacional de Ciencias Neurológicas, Lima, Perú

**Keywords:** Niclosamide, Effectiveness, Adverse events

## Abstract

**Background:**

Mass drug administration (MDA) with niclosamide (NSM) can be used to control taeniasis, the cause of neurocysticercosis. NSM is 84.3% effective against taeniasis and is considered safe as it is not absorbed from the intestinal tract. However, information on its safety and effectiveness during MDA is limited. We evaluated the effectiveness of NSM and reported adverse events (AEs) during a cysticercosis elimination program in Tumbes, Peru.

**Methods:**

Three rounds of NSM at 4-month intervals were offered to 77,397 eligible residents. We revisited all participants in their homes 72 h after each round to collect information regarding AEs. We also collected post-treatment stool samples to diagnose taeniasis after the first round, followed by a second sample at 30 days from those infected to evaluate NSM's effectiveness.

**Findings:**

During implementation, 68,751 individuals were administered at least one dose of NSM (mean age 29 years, SD 20; 52% male), and 65,551 (95.3%) were visited post-treatment. 988 (1.5%) reported experiencing at least one AE. Almost all AEs (99.2%) were of mild intensity, with no severe AEs recorded. Of 211 participants diagnosed with taeniasis, 188 provided a follow-up stool sample 30-days after treatment and 141 were cured (treatment effectiveness 75.0%). Older age and higher coproantigen levels were significantly associated with treatment failure.

**Interpretation:**

MDA with NSM is safe in *Taenia solium* endemic settings. However, the effectiveness following one dose is lower than expected, which suggests additional treatment may be necessary to enhance the infection control efforts.

**Funding:**

The 10.13039/100000865Bill and Melinda Gates Foundation.

**Research in context**

**Evidence before this study**

Controlling the transmission of *Taenia solium* (pork tapeworm) through the implementation of safe and effective strategies is vital for alleviating the health, economic, and social burdens imposed by this parasite in endemic areas. Niclosamide (NSM) mass drug administration (MDA), or delivering NSM to all people living in a community or geographic area, regardless of infection status, is a key strategy to target taeniasis. While NSM is considered safe and effective, evidence regarding its use during MDA implementation is still lacking. We searched PubMed, Google Scholar, WHO bulletins, and Cochrane databases for articles published up to 2022 using the search terms “mass drug administration,” “mass treatment,” “niclosamide,” “anthelmintic,” “safety,” “adverse events,” “adverse effects,” “adverse reactions,” “effectiveness,” “taeniasis,” and “[neuro]cysticercosis”; in total, 15 articles were identified.

NSM has proven effective in treating taeniasis, with selective chemotherapy studies estimating 77.9% to 94.8% effectiveness and MDA studies demonstrating lower taeniasis prevalence in intervention compared to control communities. While no adverse events were reported during NSM MDAs, gastrointestinal events were reported in selective chemotherapy studies and in the World Health Organization (WHO) database of adverse drug reactions (ADR). Overall, safety monitoring systems often lacked detail and relied on passive reporting systems, highlighting the need for improved monitoring, as emphasized by the 2021 Pan American Health Organization (PAHO) guidelines for evidence-based preventative chemotherapy for *T. solium*.

**Added value of this study**

This study presents the largest and most comprehensive evaluation of NSM's safety and effectiveness during MDA in a *T. solium* endemic setting. We actively monitored over 65,000 residents living in Northern Peru using a standardized safety questionnaire, which was administered during home visits after each round of NSM administration. Additionally, the effectiveness of NSM was assessed for a large subpopulation who provided stool samples following their first dose of NSM. In this assessment, we used diagnostic tools that were highly sensitive and specific for identifying taeniasis, thus yielding more accurate estimates of effectiveness.

**Implications of all the available evidence**

Our findings were relatively consistent with prior evidence, affirming that NSM is a safe and effective medication for MDAs in *T. solium* endemic areas. Adverse events were rare, non-serious, and primarily gastrointestinal. Importantly, there were no reports of any serious neurologic adverse events indicative of inflammation resulting from a drug interaction with viable *T. solium* brain cysts. This evidence supports recommendations for using NSM during MDA in regions where undiagnosed cases of neurocysticercosis are likely to be present.

Despite NSM's impressive safety profile during MDA, its single-dose effectiveness was lower than expected, particularly among older individuals and those with higher antigen levels in their initial post-treatment stool sample. These factors may limit the medication's ability to successfully treat infections at its current recommended regimen, warranting the need for further research to assess different NSM treatment approaches in a large population setting.

## Introduction

The zoonotic cestode, *T. solium* (i.e., pork tapeworm), causes widespread disease in pigs and humans in the forms of cysticercosis and taeniasis. Cysticercosis refers to the systemic infection with metacestodes (i.e., larval stage) that occurs in both humans and pigs. With cysticercosis, cysticerci develop throughout different tissues of the body, most notably in skeletal muscles and the central nervous system (CNS). Among humans, cysticercosis of the CNS, i.e., neurocysticercosis (NCC), is a leading cause of acquired epilepsy in *T. solium* endemic settings, accounting for approximately one-third of all seizure disorders.^1^ In Latin America, an estimated 1.3 million people have seizures attributable to NCC.^2^

Taeniasis refers to the *T. solium* infection of the human gastrointestinal tract by the adult-stage tapeworm. This infection follows the ingestion of contaminated pork containing *T. solium* cysticerci. Ingested cysts release their scolices, which anchor onto the wall of the small intestine, where they mature into the adult tapeworm. Over a lifespan ranging from months to years, adult tapeworms continually produce proglottids, which mature, become gravid, and eventually detach from the tapeworm. Each gravid proglottid segment contains thousands of parasite eggs, which are intermittently shed in the faeces of infected individuals, thereby contaminating food, water, and other media. Subsequent inadvertent ingestion of these eggs can result in NCC.

Treatment of taeniasis is critical for interrupting the *T. solium* lifecycle and controlling transmission, yet it is frequently overlooked. Because taeniasis typically presents with no more than mild symptoms, if any, many infections are undetected and untreated. These unidentified, persistent infections increase the community's risk of NCC through egg distribution in the form of contaminated food or water and subsequent ingestion by other community members. Recognising the need to control cysticercosis, the World Health Organization (WHO) recommends efforts to implement high-quality and safe interventions in areas endemic to *T. solium*, such as mass drug administration (MDA).^3^ This strategy involves delivering an intestinal anthelminthic medication (e.g., niclosamide (NSM), praziquantel (PZQ), or albendazole (ALB)) to all eligible community members, irrespective of infection status.^3^^,^^4^ It is a well-established and logistically feasible approach for providing medications to individuals living in communities with limited healthcare access.

The drug safety profile is an important consideration in selecting an anthelminthic for MDA. In regions where *T. solium* is endemic, both NCC and taeniasis infections may co-occur within individuals. Further, those without taeniasis may have NCC, putting them at risk for side effects from the drug without any direct benefit. With the co-occurrence of these two related but distinct infections, the use of systemically absorbed medications such as PZQ or ALB for MDA targeted at taeniasis can inadvertently cause the death of viable CNS cysts, resulting in a potentially life-threatening inflammatory response. NSM does not impose the same risk as it is only minimally absorbed from the intestinal tract. Previously, severe neurological adverse events following treatment with PZQ^5^, ^6^, ^7^, ^8^ or ALB^9^, ^10^, ^11^, ^12^ have been observed among people who were later identified to have NCC. Despite these events, safety surveillance systems for anthelminthic medications remain limited in areas where *T. solium* is endemic.

Treatment with a single dose of NSM for taeniasis has demonstrated an estimated effectiveness of 84.3% (95% Confidence Interval (CI): 64.4%, 99.3%) among those undergoing selective chemotherapy.^4^ Side effects related to NSM are uncommon and are mostly reported to be mild and primarily related to gastrointestinal upset.^13^ However, despite the known safety and effectiveness of NSM for targeted individual treatment,^14^, ^15^, ^16^, ^17^, ^18^, ^19^, ^20^, ^21^, ^22^ there is little published information about the safety and effectiveness of using NSM as a community-wide treatment strategy.^13^^,^^23^

In Peru, a *T. solium* elimination project was conducted between 2004 and 2010.^24^ In the third phase of implementation, three rounds of mass treatment with NSM were offered to eligible residents living in peri-urban and rural communities in the Tumbes Region. During this phase, 84.7% of residents received at least one dose of NSM.^24^ In this study, our primary objective is to describe the safety outcomes following MDA with NSM. Additionally, we will describe participant characteristics related to the effectiveness of NSM in treating taeniasis.

## Methods

### Study design and population

We used a cross-sectional study design to evaluate the safety and effectiveness of MDA with NSM for *T. solium* taeniasis. This evaluation was nested within a larger Cysticercosis Elimination Demonstration Program, which is described in detail elsewhere (Figure S1).^24^ In brief, the program included a series of interventions targeting the control of *T. solium* infections among both humans and pigs in Tumbes, Peru. During the final phase of the program in 2009–2010, residents located in all 107 rural and peri-urban villages (population 81,170) were offered oral NSM during three rounds of MDA. Four-month intervals separated each round of NSM MDA: round 1 (June–August 2009), round 2 (October 2009–February 2010), and round 3 (February–May 2010). The study was approved and registered by the Peruvian National Institute of Health (resolution 066-2005-J-OPD/INS, Code INS_ 002-05). Ethical approval for the study was obtained from the main institutional review board at Universidad Peruana Cayetano Heredia (FWA00000525, SIDISI 03101).

Prior to the MDA activities, a census was conducted in all study villages. Children younger than two years of age or less than 11 kg (kg) were excluded from treatment, as were pregnant or breastfeeding individuals (determined by urine test or self-report). All remaining 77,397 residents were eligible to participate. Informed consent was obtained during household visits with witnessed signatures on forms. Parental consent was secured for minors, while participants who were unable to read or write had a literate adult co-sign.

### Procedures

#### Niclosamide procedures

Niclosamide (500 mg) tablets (STEROP SA, Belgium) were used and offered to eligible residents. For those who accepted treatment, the drug was administered in a single oral dose: two grams for adults, one gram for children older than six years of age, and approximately 50 mg/kg for children 2–6 years old. Study personnel ground all tablets with a mortar and pestle at the time of treatment, and the resulting powder was mixed with fruit juice for ingestion. A single dose of NSM was orally administered under direct supervision at participants' homes during each round. Medications were not left unattended at homes. If participants were unavailable, three additional household visits were conducted during the same round to attempt to administer NSM.

#### Safety surveillance

Participants who accepted NSM were monitored for adverse events (AE) immediately following treatment and during the initial 72 h. Study personnel, trained to recognize and assess NSM-related AE prior to each MDA round, returned to all participants’ homes 72 h after treatment to administer a standardized adverse events questionnaire (AEQ). Individuals who reported any AE during follow-up were evaluated by our study physician for medical care and monitored until their symptoms resolved. Additionally, quality control of reported AE was ensured by the study physician and field coordinator, who revisited participants at their homes.

During the evaluation, the study physician confirmed the relatedness to NSM and severity of all reported AEs. Relatedness was assessed based on temporal association with NSM, its known side effects, and absence of alternative explanations. AE meeting these criteria were considered related to NSM, while those not meeting the criteria were unrelated. Severity was classified as three categories (mild, moderate, and severe). Mild AEs were defined as events causing minimal, transient discomfort that did not interfere with activities of daily living and did not require intervention, such as a mild headache. Moderate AEs were defined as events that caused sufficient discomfort to interfere with activities of daily living and may have required symptomatic intervention (e.g., nausea requiring treatment with an antiemetic). Severe AEs were those that completely prevented participation in activities of daily living and may have required more invasive intervention, treatment, or hospitalization (e.g., a seizure).

Data from all three rounds of the AEQ were aggregated into a final dataset. Most residents (82%) completed all three surveys, with the remaining participating in one or two rounds only. The primary outcome was the occurrence of any AE reported 72 h after NSM treatment. Participants who reported any NSM-related AE during any of the three treatment rounds were categorized as having an AE. Those without any reports were categorized as having none. Additionally, we characterized the severity of AE (mild/moderate/severe), type of AEs, the cumulative number of AEs reported (1 AE/2 AEs/≥ 3 AEs), and those who reported AEs across multiple rounds (one round, only/two rounds/three rounds).

#### NSM effectiveness

Following the first round of NSM treatment, stool samples were collected from all participants 24 h post-administration to establish a baseline. Fifty-millilitre aliquots of stool were preserved in 5% formol and then shipped to the Universidad Peruana Cayetano Heredia for diagnosis of *Taenia* sp. Samples were evaluated using three methods: 1) visual inspection of the whole stool for the presence of proglottids or scolexes, 2) microscopic inspection after 24-h spontaneous sedimentation to detect the presence of tapeworm eggs, and 3) coproantigen enzyme-linked immunoassay (coAg ELISA) to detect the presence of tapeworm antigens.^25^^,^^26^ Samples were classified as positive for *T. solium* taeniasis if parasite material was identified or if antigen levels reached a percentage of positivity (PP) ≥ 20 (defined as the optical density (OD) of the sample/OD of a strong positive control x 100). Otherwise, a sample was classified as negative for infection. Individuals diagnosed with taeniasis from their baseline sample submitted a second stool sample approximately 30 days post-treatment.

Our secondary outcome focused on NSM treatment effectiveness among baseline-positive participants. Effectiveness was defined as treatment success or those who tested negative for taeniasis approximately 30 days after NSM treatment, indicating infection clearance. Conversely, treatment failure included cases that remained positive for taeniasis.

### Statistical analysis

Statistical analyses were conducted using Stata SE17 and R version 4.2.2. In our descriptive safety analysis, we reported the prevalence and 95% confidence intervals (CI) for any adverse event related to NSM during the intervention period. The prevalence of any AEs was estimated as the proportion of treated individuals who reported experiencing one or more AE after being treated with NSM, divided by the total number of treated individuals who responded to the AEQ during the implementation period.

Participant characteristics were reported by any AE. We reported frequencies (proportions) for all categorical variables, including participant sex (male/female), rurality (rural/peri-urban), access to public water source (yes/no), public electricity (yes/no), household sanitation (bathroom/latrine/none), livestock rearing (yes/no), participation in NSM mass treatment (one/two/three rounds), and diagnosis with taeniasis following the first round of treatment (yes/no). Means (standard deviations [SD]) and medians (ranges) were reported for all continuous variables, including age (years) and number of household members. The severity, type, number of AE experienced per participant, and AEs across multiple rounds were summarized among those who experienced any AE related to NSM overall and stratified by sex.

For participants diagnosed with taeniasis following the first round of treatment, we reported the effectiveness (95% CI) of NSM in successfully treating taeniasis. Effectiveness was estimated as the proportion of taeniasis cases with negative stool samples 30 days after NSM treatment. Measures of central tendency were reported for the variable age and baseline antigen levels, while measures of distribution were reported for the categorical variables’ sex, baseline antigen levels (≥median/< median) and baseline parasite burden was reported by treatment outcome (failure/success). To assess variables associated with treatment failure among individuals diagnosed with taeniasis, we used a bivariate log-binomial regression model to estimate unadjusted prevalence ratios and their 95% CI for the relationship between treatment failure and each selected independent variable. Subsequently, we performed a multivariable regression analysis that included age, sex, and baseline coproantigen levels (≥median/< median) to report adjusted prevalence ratios and their 95% CIs.

### Missing data

We conducted a descriptive analysis of the characteristics of participants who were excluded from our final analytic samples. This assessment included those who were excluded due to nonresponse to the AEQ or those without a stool sample for the safety and effectiveness analyses, respectively. This was conducted for participants available during each round and for those aggregated across all three rounds (Tables S1–S3). Additionally, we performed multiple imputations and best-worst case scenarios as sensitivity analyses (Tables S4–S6).

### Role of the funding source

The Bill and Melinda Gates Foundation had no role in the study design, data analysis, interpretation, or preparation of the report.

## Results

### Safety analysis

**Fig. 1:**
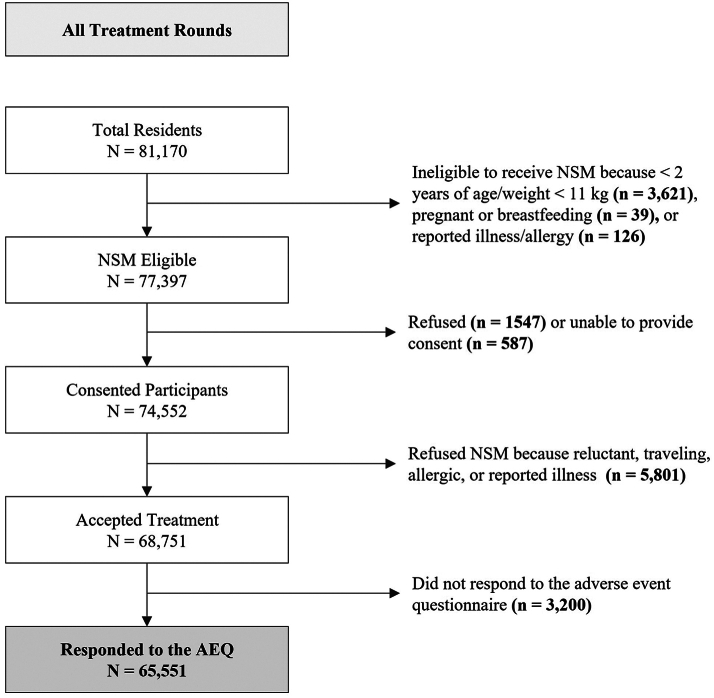
Flow diagram of all residents from 107 villages in Tumbes, Peru who participated in any of the three rounds of mass treatment with oral niclosamide (NMS) and responded to the adverse even questionnaire 72 h after accepting treatment, June 2009–April 2010.

Among the 77,397 residents who were eligible to participate, 68,751 (88.8%) residents consented and accepted at least one round of NSM treatment (Fig. 1). Within this population, 65,551 (95.3%) were successfully contacted 72 h after they received NSM and completed the AEQ.

Across all rounds of the MDA, 1088 participants reported symptoms during the AEQ, of which 988 were determined to be AE related to NSM, resulting in a prevalence of 1.5% (95% CI: 1.4%, 1.6%). Residents who experienced any related AE were on average older, more likely to be female, and more likely to have participated in all three rounds of NSM treatment compared to those who reported no AE (Table 1). Distributions of all other household characteristics were similar across outcome groups. Additionally, among the 41,398 participants who provided a stool sample following the first round of NSM MDA (Figure S2), the distribution of any AE did not differ among those diagnosed with taeniasis. AE stratified by round and unrelated AE are detailed in the Supplementary Materials (Table S7–S9).

**Table 1:** Characteristics according to the occurrence of any adverse event during niclosamide mass treatment implementation among 65,551 residents living in Tumbes, Peru between 2009 and 2010.

	Any adverse event	No adverse event
	N = 988	N = 64,563
	n (%)	n (%)
**Age (Years), mean (SD)**	34.0 (18.7)	29.7 (19.6)
**Age categories (Years)**		
<20	244 (24.7%)	24,454 (37.9%)
20-39	386 (39.1%)	21,206 (32.8%)
40-59	259 (26.2%)	13,216 (20.5%)
60+	99 (10.0%)	5687 (8.8%)
**Participant sex**		
Male	296 (30.0%)	33,090 (51.3%)
Female	692 (70.0%)	31,473 (48.7%)
**Household location**		
Rural	561 (56.8%)	32,038 (49.6%)
Peri-urban	427 (43.2%)	32,525 (50.4%)
**Number of household members, mean (SD)**	4.18 (1.74)	4.24 (1.66)
**Household access to public water source**		
Yes	436 (44.1%)	24,509 (38.0%)
No	552 (55.9%)	40,054 (62.0%)
**Household Access to Public Electricity**		
Yes	188 (19.0%)	9485 (14.7%)
No	800 (81.0%)	55,078 (85.3%)
**Household sanitation**		
Bathroom	277 (28.0%)	23,946 (37.1%)
Latrine	416 (42.1%)	24,318 (37.7%)
None	295 (29.9%)	16,299 (25.2%)
**Household raises livestock**		
Yes	653 (66.1%)	42,929 (66.5%)
No	335 (33.9%)	21,634 (33.5%)
**Number of rounds of NSM treatment**		
1 round	133 (13.5%)	11,444 (17.7%)
2 rounds	256 (25.9%)	19,028 (29.5%)
3 rounds	599 (60.6%)	34,091 (52.8%)
**Diagnosed with Taeniasis**^a^		
Infected	3 (0.3%)	205 (0.3%)
Not infected	667 (67.5%)	39,816 (61.7%)
Missing Data	318 (32.2%)	24,542 (38.0%)

a Taeniasis was diagnosed based on stool samples collected 24 h after the first round of NSM mass treatment, only. Of the 65,551 residents who received at least one dose of NSM during any of the three rounds, 13,308 (20%) did not accept or receive treatment during the first round, 5153 (8%) did not respond to the AEQ, and 6399 (10%) did not provide a stool sample.

Among the 988 participants who reported any AE related to NSM treatment, almost all (99.2%) events were of mild intensity (Table 2). Moderate AEs were experienced by eight participants (0.8%) and included headache, rash, or bitter taste. Overall, abdominal discomfort was the most commonly reported event type (56.4%), followed by headache (24.6%), tongue numbness (14.3%), and diarrhoea (13.8%). The majority of participants reported only one adverse event following treatment; however, 38.0% reported two AEs and 11.5% reported three or more AEs. Most of those who reported multiple AEs reported them within a single round. Among the 18 participants who reported adverse events across multiple treatment rounds, all were female (Table S10). Notably, abdominal discomfort, headache, tongue numbness, and rash were identified as recurring adverse events among these participants (Table S11).

**Table 2:** Adverse events reported among participants who responded to the adverse event questionnaire (AEQ) following multiple rounds of niclosamide (NSM) mass treatment in Tumbes, Peru, 2009-2010.

Adverse event	Any adverse event
	N = 988
	n (%)
**Severity of adverse event**	
Severe	0 (0.0%)
Moderate	8 (0.8%)
Mild	980 (99.2%)
**Type of adverse event**	
Abdominal discomfort	557 (56.4%)
Headache	243 (24.6%)
Tongue numbness	141 (14.3%)
Diarrhea	136 (13.8%)
Nausea	98 (9.9%)
Watery stool	80 (8.1%)
Vomiting	53 (5.4%)
Dizziness	50 (5.1%)
Rash	33 (3.3%)
Other types of pain	30 (3.0%)
Discomfort	29 (2.9%)
Pruritus	16 (1.6%)
Constipation	12 (1.2%)
Abdominal distension	9 (0.9%)
Epigastric burning	6 (0.6%)
Bitter Taste	4 (0.4%)
Fever	4 (0.4%)
Other	104 (10.5%)
**All adverse events reported, count**	
Mean (SD)	1.64 (0.78)
Median (Min, Max)	1.00 (1.00, 6.00)
**All adverse event reported, categorical**	
1	502 (51.2%)
2	372 (38.0%)
3+	114 (11.5%)
**Any recurrent adverse event(s)**	
None—AE occurred in one round only	970 (98.2%)
AE occurred in two rounds	17 (1.7%)
AE occurred in three rounds	1 (0.1%)

### NSM effectiveness

24 h following the first round of treatment, stool samples were collected for 41,398 (86.1%) of 48,099 participants who accepted NSM treatment. Among these participants, 211 (0.5%) were identified as positive for taeniasis and 188 provided a stool sample approximately 30 days (median: 32 days, IQR: 24–36 days) after treatment (Figure S1).

Among the 188 people with evidence of taeniasis, 75.0% (95% CI 68.4%, 80.6%) tested negative for infection 30 days post-treatment (i.e., treatment success). Those who tested positive for taeniasis at 30 days post-treatment (i.e., treatment failure) were on average older and had higher coproantigen levels at 24 h post-treatment compared to those who successfully cleared the infection (Table 3).

**Table 3:** Demographic, stool characteristics, and log-binomial regression models for treatment failure among taeniasis-positive participants, Tumbes, Peru, June–August 2009.

	Overall	Treatment failure	Treatment success	Bivariate models	Multivariable model
	N = 188	N = 47	N = 141	N = 188	N = 188
	n (%)	n (%)	n (%)	PR (95% CI)	aPR (95% CI)
**Age (Years), mean (SD)**	32.2 (17.5)	36.8 (18.8)	30.7 (16.8)	1.01 (1.00, 1.03)	1.02 (1.00, 1.03)
**Sex**					
Male	85 (45.2%)	23 (48.9%)	62 (44.0%)	1.16 (0.65, 2.06)	1.21 (0.68, 2.15)
Female	103 (54.8%)	24 (51.1%)	79 (56.0%)	ref	ref
**Coproantigen levels (categorical)**^a^					
≥median (30.3 PP)	94 (50.0%)	34 (72.3%)	60 (42.6%)	2.62 (1.42, 5.14)	2.73 (1.47, 5.36)
<median	94 (50.0%)	13 (27.7%)	81 (57.4%)	ref	ref
**Coproantigen levels (PP)**^a^					
Mean (SD)	50.6 (51.5)	73.0 (58.5)	43.2 (46.8)	1.01 (1.00, 1.01)	
Median (min, max)	30.3 (1.6, 251.0)	43.0 (15.3, 234.0)	27.1 (1.6, 251.0)	ref	
**Taeniasis present with another parasite**^a^					
Yes	109 (58.0%)	26 (55.3%)	83 (58.9%)	0.90 (0.51, 1.61)	
No	79 (42.0%)	21 (44.7%)	58 (41.1%)	ref	

**Abbreviations:** standard deviation (SD), niclosamide (NSM), percentage of positivity (PP), prevalence ratio (PR), confidence interval (CI), adjusted PR (aPR).

a Based on stool sample collected 24 h following NSM treatment. Treatment failure was defined as participants positive for taeniasis at 30-day follow-up, while treatment success was defined as participants negative for taeniasis at 30-day follow-up. Prevalence ratios (PR) and their 95% confidence interval (CI) were estimated using a simple, bivariate log-binomial regression model to represent the unadjusted association between each independent variable (age, sex, coproantigen levels, and taeniasis + another parasite) and the occurrence of treatment failure. Continuous coproantigen levels and age contain 1 in the CI, suggesting borderline significance in the bivariate model. Continuous coproantigen levels are non-parametric, thus a categorical variable was created based on the group's median coproantigen level at baseline (median [Q1, Q3]: 30.3 [22.1, 63.2]).The multivariable model was adjusted for age, sex, and categorical coproantigen levels.

From our bivariate and multivariable regression analysis, only age and baseline coproantigen levels (median) were significantly associated with treatment failure (Table 3). In the multivariable model, for every 1-year increase in age, the prevalence of treatment failure is expected to increase by approximately 2% (95% CI: 1.00, 1.03). The prevalence of 30-day treatment failure was almost 3-fold greater among those with coproantigen levels above the median (30.3 PP) based on their initial stool sample when compared to the prevalence among those with lower coproantigen levels (aPR: 2.73; 95% CI: 1.47, 5.36).

### Missing data analysis

Among those who accepted treatment, participants who did not respond to the AEQ (N = 3200; 4.7%) were excluded. Those who were excluded had a younger mean age and were more likely to be male and not raise livestock when compared to those who responded to the AEQ (Table S1).

For taeniasis screening following the first round of NSM treatment, 6700 participants did not provide a stool sample. Those who were excluded had a younger mean age and were more likely male when compared to those who provided a stool sample (Table S2). Among those who were positive for taeniasis, 10.9% were missing a follow-up stool sample. Those without a second stool sample were more likely male and from a rural location when compared to those who provided a stool sample (Table S3).

The estimated prevalence of taeniasis, adverse event outcomes, and NSM effectiveness remained stable during missing data imputation (Table S5). Further, sensitivity analyses considering various infectious status scenarios among individuals missing follow-up stool samples yielded effectiveness estimates ranging from 66.8% to 77.7% (Table S6).

## Discussion

In this large-scale, community-based study in Northern Peru, we demonstrated the safety and effectiveness of NSM mass treatment for *T. solium* taeniasis. Overall, we observed a low prevalence (1.5%) of reported AEs among the 65,551 participating residents. In addition to this low prevalence, most AEs, when they did occur, were classified as mild and gastrointestinal in origin, and no serious adverse events were reported. In particular, there were no reports of seizures or other serious neurologic AEs that might be attributable to intracranial inflammation resulting from a drug interaction with viable *T. solium* brain cysts. This evidence supports the safety of NSM for use in MDA in areas where undiagnosed cases of NCC are likely to exist. Beyond the impressive safety profile, among participants with taeniasis, 75.0% successfully cleared their infection within 30 days following the first round of treatment.

To our awareness, this study is the largest safety and effectiveness assessment of NSM used in MDA for controlling *T. solium*. Prior MDAs using NSM were largely carried out in either Peru or Guatemala as part of a field trial,^27^, ^28^, ^29^ a community-wide treatment strategy,^23^ or in prior phases of this elimination program.^24^ In these studies, no AE outcomes were reported. Gastrointestinal AEs following NSM administration have been previously captured by safety surveillance systems^30^^,^^31^ or during other studies evaluating the use of NSM to treat taeniasis.^14^^,^^15^^,^^32^

Notably, female participants were more likely to report an AE compared to male participants. While uncommon overall (0.8%; n = 8), only female participants experienced moderate AEs, and they were more likely than males to report AEs across multiple rounds. Previous studies have not addressed sex-specific drug reactions for NSM, but similar experiences have been observed for other drugs reported in international pharmacovigilance databases.^33^ These differing sex-based reactions likely stem from a variety of sex-related factors, both physiologic (hormonal, metabolic, and anatomic) and social-behavioral.^34^

While there was a low prevalence of taeniasis (0.5%) among treated participants, most (75.0%) cleared their infection 30 days after treatment. This estimate is an improvement over what was observed during phase 1 of the elimination project, where a smaller-scale mass treatment strategy was implemented. During phase 1, 63.2% (n = 24/38) were negative for taeniasis two weeks following treatment^24^ as compared to the 75.0% in the present study. Our finding is more consistent with a smaller study conducted by Bustos et al. in Peru, which reported that 77.9% (n = 86) of participants were cured 90 days after NSM treatment.^22^ In contrast, Varma et al. in India estimated a higher effectiveness (94.8%; n = 38) 90 days following NSM treatment,^15^ but this study only used microscopy for taeniasis diagnosis, a method requiring direct visualisation of taenia eggs. Microscopy, while commonly used, has a low sensitivity (52.5%) due to the intermittent shedding of eggs.^16^ In the elimination project and the Bustos study, both microscopy and the coproantigen ELISA immunoassay (sensitivity: 96%; specificity: 100%) were used, which may account for similar estimates in the proportion cured.^26^^,^^35^ Importantly, the use of this immunoassay enhances the accuracy of the treatment outcome as those with persistent infections will remain positive during temporary cessation of egg shedding.

Interpretation of our results should be placed within the limitations of our study. First, we estimated the prevalence of any AE among a population treated with NSM. We were unable to obtain information on background rates of disease or the occurrence of events among an untreated population. This limitation prevented us from distinguishing between safety concerns and events that coincidentally occurred in temporal association. This limitation hindered our ability to assess causal associations between NSM and the AEs. Nonetheless, a physician evaluated and monitored all reported AEs to better determine those likely related to the treatment.

Second, we considered the potential for clustering of responses by individuals across survey rounds, as participants who experience an AE might be more likely to experience another following subsequent NSM rounds. However, clustering was minimal as only 0.03% participants (n = 18) experienced AEs in multiple rounds.

Third, this analysis could be impacted by nonresponse. Male participants were more likely to be excluded from the safety analysis. Given that male participants were less likely to report any AE among the analytic sample, we expect that we may have slightly overestimated the prevalence of any AE in the target population. For our NSM effectiveness analysis, participants who were younger, male, and living in a rural community were more likely to be excluded due to nonresponse. The demographics of the excluded population were similar to those not screened for taeniasis and those without a follow-up stool sample. We do not suspect these individuals were systematically excluded based on their taeniasis infection status. Further, given the low prevalence of taeniasis within this population, we expect minimal, if any, influence from this selection bias on our results. These assumptions were confirmed by imputed estimates generated in the sensitivity analyses.

Fourth, participants were screened for taeniasis following the first round of MDA only. As such, treatment effectiveness results reflect infection clearance following the first rather than all three rounds of MDA. Additionally, infection clearance was measured approximately 30 days after treatment. It is plausible that we may have observed a larger or smaller estimated effectiveness if additional stool samples were collected at 60- or 90-days post-treatment, as it would potentially take weeks or months for any persistent tapeworms to regenerate their strobila and shed gravid proglottids or eggs following a dose of treatment.

Key strengths of this study include the large, population-based sample, the highly sensitive diagnostics used to detect taeniasis, and the active and systematic safety surveillance. Further, as part of our safety evaluation, all study participants were visited in their homes 72 h after treatment. Through the study's procedures, we feel there is a low likelihood that adverse events related to NSM were missed. The extent of surveillance provides strong empirical evidence about the safety of using NSM for mass treatment of taeniasis in Peru, especially in a population unscreened for NCC. Finally, the representativeness of this study's findings was bolstered by our comprehensive census-based approach, capturing a large extent of the eligible population living in rural and peri-urban communities in Tumbes, Peru during implementation.

In summary, the morbidity and mortality associated with *T. solium* infections, most notably NCC, compels the ongoing effort to define the safest and most effective protocols for mass treatment of taeniasis. In this study, NSM demonstrated a strong safety profile among the 68,751 treated individuals who were unscreened for NCC. Despite its safety, one dose of NSM showed a lower effectiveness at 30 days post-treatment than anticipated, especially among those who were older and those with higher antigen levels. These characteristics may reduce the medication's ability to control transmission of disease with a single dose. Given NSM's impressive safety profile in a population where NCC is endemic, these findings warrant further research to evaluate strategies focused on the effectiveness of different NSM treatment regimens in a large population setting.

## Contributors

HHG and RHG contributed to study conceptualization, RG, RV, CM, AGL, LMM, and GEG to data curation, AEF, RHG, and HHG to supervision, MTW, RG, and SEO to formal analysis, MTW, SEA, RG, SEO, and HHG to writing the original draft, review, and editing, and MTW, SEA, RG, PV, and SEO accessed and verified the underlying raw data in the manuscript. All authors reviewed the final manuscript, had full access to all the data in the study, and accepted responsibility to submit for publication. HHG had final responsibility to submit for publication.

Other members of the Cysticercosis Working Group of Peru (CWGP) include Manuela Verastegui, PhD, Javier Bustos, MD, MS, MPH, PhD; Mirko Zimic, PhD (coordination board); Isidro Gonzalez, MD; Herbert Saavedra, MD; Sofia Sanchez, MD, MS; Manuel Martinez, MD (Instituto Nacional de Ciencias, Neurologicas, Lima, Peru); Yesenia Castillo, MSc; Luz Toribio, MSc; PhD; Gianfranco Arroyo, DVM, PhD; Miguel A. Orrego, MS, PhD; Nancy Chile, PhD; Holger Mayta, PhD; Monica Pajuelo, PhD; Saul Santivañez, MD (Universidad Peruana Cayetano Heredia, Lima, Peru); Eloy Gonzalez-Gustavson, DVM, MS, PhD; Luis Gomez, DVM, PhD; Cesar M. Gavidia, DVM, MPH, PhD; Ana Vargas-Calla, DVM, MS; Maria T. Lopez, DVM, PhD (Universidad Nacional Mayor de San Marcos, Lima, Peru); Theodore Nash, MD (National Institute of Allergy and Infectious Diseases, National Institutes of Health, Bethesda, Maryland, United States of America); Sukwan Handali, MD, MS, PhD; John Noh (Center for Diseases and Control, Atlanta, Georgia, United States of America); and John Friedland, PhD (St George, University of London, United Kingdom).

## Data sharing statement

Data can be made available upon reasonable request by contacting the corresponding author.

## Declaration of interests

RG, PV, CM, and LMM were partially supported by the US National Institutes of Health, the Fogarty International Center (TW001140), AGL was supported by Emerge, the Emerging Diseases Epidemiology Research Training of the US National Institutes of Health, the Fogarty International Center (D43 TW007393), and MTW was supported by the National Center for Advancing Translational Sciences of the US National Institutes of Health under award number (TL1TR00237). HG was supported by a Wellcome Trust International Senior Research Fellowship in Public Health and Tropical Medicine. All authors declare no conflicts of interests.

## Appendix A Supplementary data

Translated Abstract

Supplementary Figures and Tables

Members-table
