# Supplementary material for: Mass chemotherapy with niclosamide for the control of Taenia solium: population-based safety profile and treatment effectiveness
Source: Lancet Reg Health Am. 2024 Aug 30;38:100876. doi: 10.1016/j.lana.2024.100876 (PMC11402444; doi:10.1016/j.lana.2024.100876)
Supplement: Translated Abstract [file mmc1.docx]

**Editor note:** *This translation in Spanish was submitted by the authors and we reproduce it as supplied. It has not been peer reviewed. Our editorial processes have only been applied to the original abstract in English, which should serve as reference for this manuscript*

**RESUMEN**

**ANTECEDENTES:** La administración masiva de medicamentos (MDA) con niclosamida (NSM) se puede utilizar para controlar la teniasis, la causa de la neurocisticercosis. La NSM tiene una eficacia del 84-3% contra la teniasis y se considera segura ya que no se absorbe en el tracto intestinal. Sin embargo, la información sobre su seguridad y eficacia durante la MDA es limitada. Este estudio evaluó la efectividad de la NSM y reporta los eventos adversos (EA) durante un programa de eliminación de cisticercosis en Tumbes, Perú.

**MÉTODOS:** Se ofrecieron tres rondas de NSM a intervalos de 4 meses a 77.397 residentes elegibles. Todos los participantes fueron visitados en sus hogares 72 horas después de cada ronda para recopilar información sobre los EA. También recolectamos muestras de heces post-tratamiento para diagnosticar teniasis después de la primera ronda, seguidas de una segunda muestra de los individuos infectados a los 30 días, para evaluar la efectividad de NSM.

**RESULTADOS:** Durante la implementación, a 68.751 personas se les administró al menos una dosis de NSM (edad media 29 años, DE 20; 52 % hombres) y 65.551 (95∙3 %) fueron visitados después del tratamiento. 988 (1∙5%) informaron haber experimentado al menos un EA. Casi todos los EA (99∙2%) fueron de intensidad leve y no se registraron EA graves. De 211 participantes diagnosticados con teniasis, 188 proporcionaron una muestra de heces de seguimiento 30 días después del tratamiento y 141 se curaron (efectividad del tratamiento 75∙0%). La edad avanzada y los niveles más altos de coproantígeno se asociaron significativamente con el fracaso del tratamiento.

**INTERPRETACIÓN: L**a MDA con NSM es segura en entornos endémicos de *T. solium*. Sin embargo, la eficacia de una dosis de NSM es menor de lo esperado, lo que sugiere que puede ser necesario un tratamiento adicional para mejorar los esfuerzos de control de la teniasis/cisticercosis.

**FINANCIAMIENTO:** Fundación Bill y Melinda Gates.

**PALABRAS CLAVE:** Niclosamida, efectividad, eventos adversos.
