# Supplementary material for: Mass chemotherapy with niclosamide for the control of Taenia solium: population-based safety profile and treatment effectiveness
Source: Lancet Reg Health Am. 2024 Aug 30;38:100876. doi: 10.1016/j.lana.2024.100876 (PMC11402444; doi:10.1016/j.lana.2024.100876)
Supplement: Supplementary Figures and Tables [file mmc2.docx]

**Supplementary Materials**

**Table of Contents**

[**Figure S1.** Chronological placement of Phase 3 3](#_Toc167821368)

[**Figure S2.** Flow diagram 4](#_Toc167821369)

[**Table S1**. Demographic characteristics for all residents and by adverse event questionnaire (AEQ) response status 5](#_Toc167821370)

[**Table S2**. Demographic characteristics for all residents who participated in round 1 6](#_Toc167821371)

[**Table S3.** Demographic characteristics for all 41,399 residents who provided stool samples 7](#_Toc167821372)

[**Sensitivity Analysis Methods & Results** 8](#_Toc167821373)

[**Imputation Methods** 8](#_Toc167821374)

[**Table S4.** List of independent variables associated with missing data or study outcomes (taeniasis prevalence, niclosamide effectiveness, and any adverse events) used in imputation models. 8](#_Toc167821375)

[**Table S5.** Original and imputed outcome data 8](#_Toc167821376)

[**Scenario Methods** 9](#_Toc167821377)

[**Scenario Results** 9](#_Toc167821378)

[**Table S6.** Original and missing data scenarios for estimated niclosamide (NSM) effectiveness. 9](#_Toc167821379)

[**Any Adverse Event Stratified by Round** 10](#_Toc167821380)

[**Table S7.** Participant characteristics according to the occurrence of any adverse event during niclosamide mass treatment implementation overall and stratified by treatment round, among 65,551 residents living in Tumbes, Peru between 2009-2010. 10](#_Toc167821381)

[**Table S8.** Severity and types of adverse events reported among participants who responded to the adverse event questionnaire (AEQ) over multiple rounds of niclosamide (NSM) mass treatment and stratified by treatment round in Tumbes, Peru, 2009-2010. 11](#_Toc167821382)

[**Unrelated adverse events** 12](#_Toc167821383)

[**Table S9.** Types of adverse events unrelated to niclosamide 12](#_Toc167821384)

[**Recurrent Adverse Events during NSM Treatment Rounds** 13](#_Toc167821385)

[**Table S10.** Participant characteristics among those who reported adverse events (AE) following multiple NSM treatment rounds (n = 18) 13](#_Toc167821386)

[**Table S11.** Types of adverse events (AE) reported among participants who reported AEs following multiple NSM treatment rounds (n = 18) 13](#_Toc167821387)

[**Subgroup Analysis** 14](#_Toc167821388)

[**NSM Effectiveness** 14](#_Toc167821389)

[**Table S12.** Niclosamide (NSM) effectiveness by age category 14](#_Toc167821390)

[**Any Adverse Event** 14](#_Toc167821391)

[**Table S13.** Any adverse events by age category 14](#_Toc167821392)

[**Table S14.** Any adverse events by sex 15](#_Toc167821393)

**Figure S1.** Chronological placement of Phase 3 administration, stool collection, and safety surveillance within the *Taenia solium* Elimination Project in Tumbes, Peru.
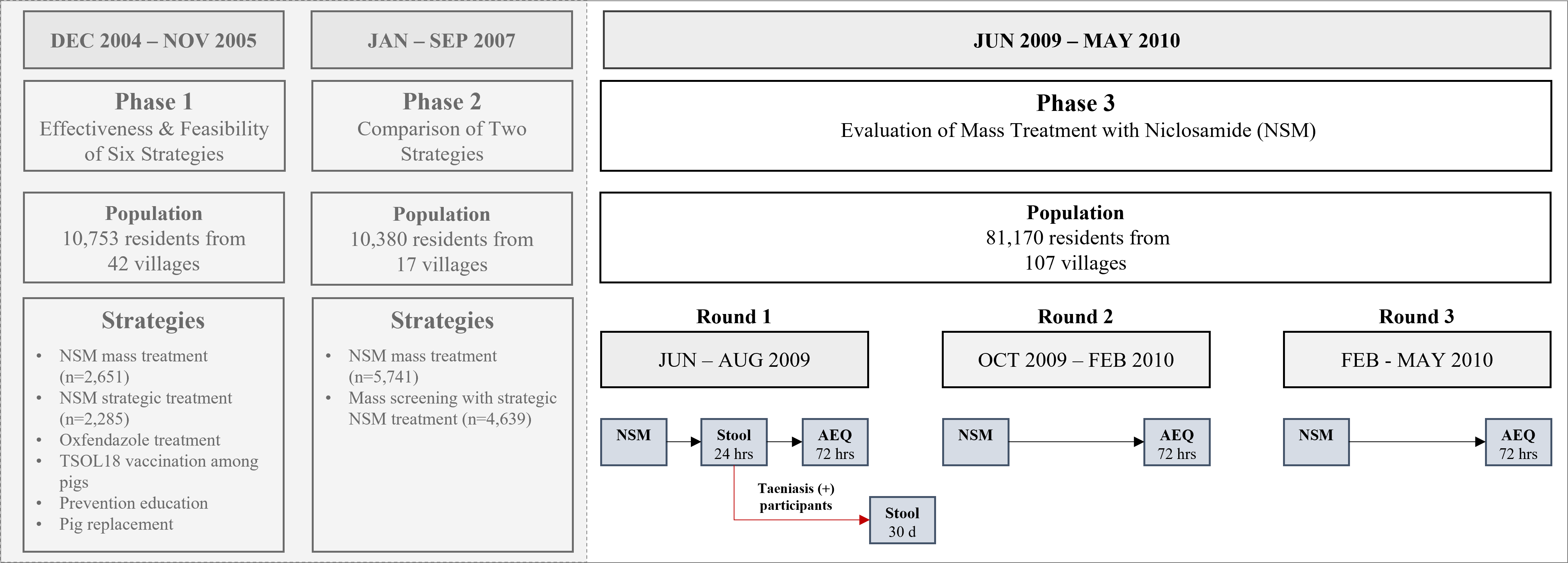


**Legend:** Phase 3 evaluated the effectiveness and safety of mass treatment with niclosamide (NSM) among 81,170 human residents from all 107 rural and peri-urban villages in Tumbes, Peru. Blue boxes represent household visits where NSM was administered, or stool samples or adverse event data were collected. Stool samples were collected from all participants 24 hours after the first round of NSM administration only. Individuals positive for taeniasis based on the first sample were contacted to provide a second sample 30 days later. Active safety surveillance was conducted among all households where NSM was administered. Participants were monitored for safety events immediately following NSM administration and revisited 72 hours to administer the adverse event questionnaire (AEQ). Other strategies implemented during Phase 3 included oxfendazole treatment and TSOL18 vaccination for pigs (not shown). The shaded area of the figure depicts timelines, overarching aim, populations, and a list of strategies implemented for Phases 1 and 2 of the Taenia solium elimination project. Strategies included mass or strategic treatment with niclosamide among humans, oxfendazole treatment and TSOL18 vaccination among pigs, prevention education, and pig replacement. Additional details about the Taenia solium Elimination Project strategies and results are reported in detail elsewhere (Garcia et al., 2016).

**Abbreviations:** niclosamide (NSM), adverse event questionnaire (AEQ), hours (hrs), days (d), diagnosed as positive for taeniasis (+).

**Figure S2.** Flow diagram of all residents from 107 villages in Tumbes, Peru who participated in any of the three rounds of mass treatment with oral niclosamide (NSM) and responded to the adverse event questionnaire 72-hours after accepting treatment by treatment round and stool sample collection, June 2009 – April 2010.


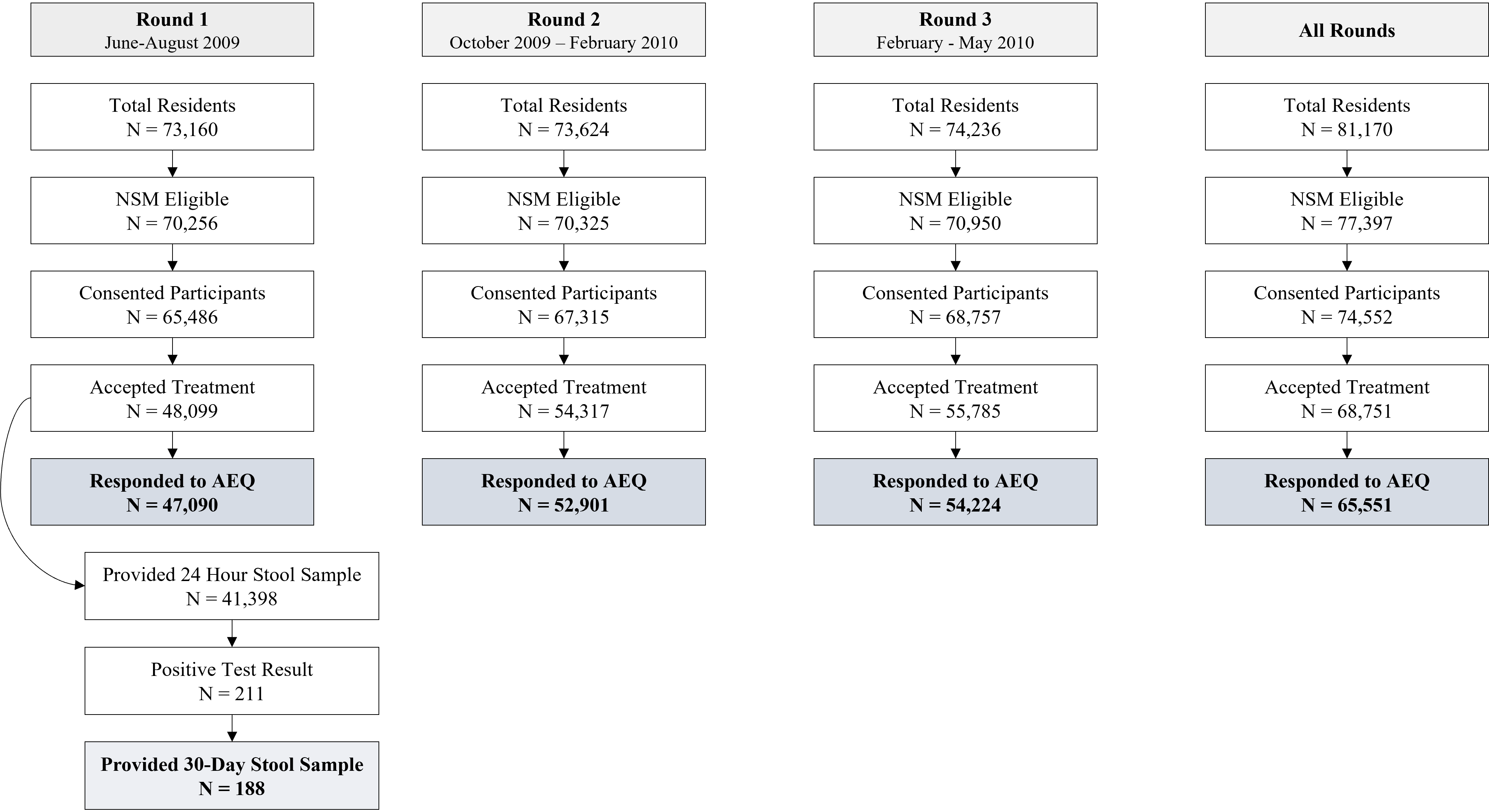


| **Table S1**. Demographic characteristics for all residents and by adverse event questionnaire (AEQ) response status among those who received niclosamide (NSM) treatment (N=68,751) | | | | | | | | |
| --- | --- | --- | --- | --- | --- | --- | --- | --- |
|  | **Accepted Treatment N = 68,751** | |  | **AEQ Response  N = 65,551** | |  | **AEQ Non-Response  N = 3,200** | |
|  | n | % |  | n | % |  | n | % |
| **Age (Years),** mean (SD) | 29.8 | 19∙5 |  | 29.7 | 19∙6 |  | 31.7 | 18∙2 |
| **Age categories (Years)** |  |  |  |  |  |  |  |  |
| < 20 | 25,529 | 37∙1% |  | 24,698 | 37∙7% |  | 831 | 26∙0% |
| 20-40 | 23,008 | 33∙5% |  | 21,592 | 32∙9% |  | 1,416 | 44∙3% |
| 40-60 | 14,159 | 20∙6% |  | 13,475 | 20∙6% |  | 684 | 21∙4% |
| 60+ | 6,055 | 8∙8% |  | 5,786 | 8∙8% |  | 269 | 8∙4% |
| **Sex** |  |  |  |  |  |  |  |  |
| Male | 35,395 | 51∙5% |  | 33,386 | 50∙9% |  | 2,009 | 62∙8% |
| Female | 33,356 | 48∙5% |  | 32,165 | 49∙1% |  | 1,191 | 37∙2% |
| **Environment** |  |  |  |  |  |  |  |  |
| Urban | 34,616 | 50∙3% |  | 32,952 | 50∙3% |  | 1,664 | 52∙0% |
| Rural | 34,135 | 49∙7% |  | 32,599 | 49∙7% |  | 1,536 | 48∙0% |
| **Water source** |  |  |  |  |  |  |  |  |
| Public network | 42,486 | 61∙8% |  | 40,606 | 61∙9% |  | 1,880 | 58∙8% |
| No public network | 26,265 | 38∙2% |  | 24,945 | 38∙1% |  | 1,320 | 41∙3% |
| **Household sanitation** |  |  |  |  |  |  |  |  |
| Bathroom | 25,367 | 36∙9% |  | 24,223 | 37∙0% |  | 1,144 | 35∙8% |
| Latrine | 25,896 | 37∙7% |  | 24,734 | 37∙7% |  | 1,162 | 36∙3% |
| Neither | 17,488 | 25∙4% |  | 16,594 | 25∙3% |  | 894 | 27∙9% |
| **Electricity** |  |  |  |  |  |  |  |  |
| Public service | 58,481 | 85∙1% |  | 55,878 | 85∙2% |  | 2,603 | 81∙3% |
| No public service | 10,270 | 14∙9% |  | 9,673 | 14∙8% |  | 597 | 18∙7% |
| **Livestock** |  |  |  |  |  |  |  |  |
| Raises animals | 45,369 | 66∙0% |  | 43,582 | 66∙5% |  | 1,787 | 55∙8% |
| Does not raise animals | 23,382 | 34∙0% |  | 21,969 | 33∙5% |  | 1,413 | 44∙2% |
| Abbreviations: standard deviation (SD), niclosamide (NSM) | | | | | | | | |

| **Table S2**. Demographic characteristics for all residents who participated in round 1 by consent status, treatment acceptance status, and stool sample provision (N=72,443) | | | | | | | | | | | | | | | | | | | | |
| --- | --- | --- | --- | --- | --- | --- | --- | --- | --- | --- | --- | --- | --- | --- | --- | --- | --- | --- | --- | --- |
|  | **Total Number of Residents N=72,443** | |  | **Number Consented N = 65,487** | |  | **Number Refused N = 6,956** | |  | **Accepted Treatment N = 48,099** | |  | **Refused Treatment N = 17,388** | |  | **Provided Stool Sample  N = 41,399** | |  | **No Stool Sample  N = 6,700** | |
|  | **n** | **%** |  | **n** | **%** |  | **n** | **%** |  | **n** | **%** |  | **n** | **%** |  | **n** | **%** |  | **n** | **%** |
| **Age (Years),** mean (SD) | 30.4 | 20∙2 |  | 30.8 | 19∙8 |  | 26.4 | 23∙2 |  | 29.3 | 19∙4 |  | 35 | 20∙3 |  | 30 | 19∙7 |  | 25.1 | 16∙7 |
| **Age categories (Years)** |  |  |  |  |  |  |  |  |  |  |  |  |  |  |  |  |  |  |  |  |
| < 20 | 26,344 | 36∙4% |  | 23,382 | 35∙7% |  | 2,962 | 42∙6% |  | 19,184 | 39∙9% |  | 4,198 | 24∙1% |  | 16,058 | 38∙8% |  | 3,126 | 46∙7% |
| 20-40 | 23,883 | 33∙0% |  | 21,726 | 33∙2% |  | 2,157 | 31∙0% |  | 14,828 | 30∙8% |  | 6,898 | 39∙7% |  | 12,446 | 30∙1% |  | 2,382 | 35∙6% |
| 40-60 | 15,089 | 20∙8% |  | 13,981 | 21∙3% |  | 1,108 | 15∙9% |  | 10,119 | 21∙0% |  | 3,862 | 22∙2% |  | 9,227 | 22∙3% |  | 892 | 13∙3% |
| 60+ | 7,127 | 9∙8% |  | 6,398 | 9∙8% |  | 729 | 10∙5% |  | 3,968 | 8∙2% |  | 2,430 | 14∙0% |  | 3,668 | 8∙9% |  | 300 | 4∙5% |
| **Sex** |  |  |  |  |  |  |  |  |  |  |  |  |  |  |  |  |  |  |  |  |
| Male | 36,995 | 51∙1% |  | 32,542 | 49∙7% |  | 4,453 | 64∙0% |  | 23,495 | 48∙8% |  | 9,047 | 52∙0% |  | 19,528 | 47∙2% |  | 3,967 | 59∙2% |
| Female | 35,448 | 48∙9% |  | 32,945 | 50∙3% |  | 2,503 | 36∙0% |  | 24,604 | 51∙2% |  | 8,341 | 48∙0% |  | 21,871 | 52∙8% |  | 2,733 | 40∙8% |
| **Environment** |  |  |  |  |  |  |  |  |  |  |  |  |  |  |  |  |  |  |  |  |
| Urban | 36,692 | 50∙6% |  | 33,248 | 50∙8% |  | 3,444 | 49∙5% |  | 24,450 | 50∙8% |  | 8,798 | 50∙6% |  | 21,232 | 51∙3% |  | 3,218 | 48∙0% |
| Rural | 35,751 | 49∙4% |  | 32,239 | 49∙2% |  | 3,512 | 50∙5% |  | 23,649 | 49∙2% |  | 8,590 | 49∙4% |  | 20,167 | 48∙7% |  | 3,482 | 52∙0% |
| **Water source** |  |  |  |  |  |  |  |  |  |  |  |  |  |  |  |  |  |  |  |  |
| Public network | 44,932 | 62∙0% |  | 40,775 | 62∙3% |  | 4,157 | 59∙8% |  | 29,818 | 62∙0% |  | 10,957 | 63∙0% |  | 25,743 | 62∙2% |  | 4,075 | 60∙8% |
| No public network | 27,511 | 38∙0% |  | 24,712 | 37∙7% |  | 2,799 | 40∙2% |  | 18,281 | 38∙0% |  | 6,431 | 37∙0% |  | 15,656 | 37∙8% |  | 2,625 | 39∙2% |
| **Household sanitation** | |  |  |  |  |  |  |  |  |  |  |  |  |  |  |  |  |  |  |  |
| Bathroom | 27,215 | 37∙6% |  | 24,584 | 37∙5% |  | 2,631 | 37∙8% |  | 17,794 | 37∙0% |  | 6,790 | 39∙1% |  | 15,371 | 37∙1% |  | 2,423 | 36∙2% |
| Latrine | 27,211 | 37∙6% |  | 24,609 | 37∙6% |  | 2,602 | 37∙4% |  | 18,273 | 38∙0% |  | 6,336 | 36∙4% |  | 15,811 | 38∙2% |  | 2,462 | 36∙7% |
| Neither | 18,017 | 24∙9% |  | 16,294 | 24∙9% |  | 1,723 | 24∙8% |  | 12,032 | 25∙0% |  | 4,262 | 24∙5% |  | 10,217 | 24∙7% |  | 1,815 | 27∙1% |
| **Electricity** |  |  |  |  |  |  |  |  |  |  |  |  |  |  |  |  |  |  |  |  |
| Public service | 62,302 | 86∙0% |  | 56,320 | 86∙0% |  | 5,982 | 86∙0% |  | 41,175 | 85∙6% |  | 15,145 | 87∙1% |  | 35,414 | 85∙5% |  | 5,761 | 86∙0% |
| No public service | 10,141 | 14∙0% |  | 9,167 | 14∙0% |  | 974 | 14∙0% |  | 6,924 | 14∙4% |  | 2,243 | 12∙9% |  | 5,985 | 14∙5% |  | 939 | 14∙0% |
| **Livestock** |  |  |  |  |  |  |  |  |  |  |  |  |  |  |  |  |  |  |  |  |
| Raises animals | 48,012 | 66∙3% |  | 43,621 | 66∙6% |  | 4,391 | 63∙1% |  | 32,458 | 67∙5% |  | 11,163 | 64∙2% |  | 28,062 | 67∙8% |  | 4,396 | 65∙6% |
| Does not raise animals | 24,431 | 33∙7% |  | 21,866 | 33∙4% |  | 2,565 | 36∙9% |  | 15,641 | 32∙5% |  | 6,225 | 35∙8% |  | 13,337 | 32∙2% |  | 2,304 | 34∙4% |
| Abbreviations: standard deviation (SD), niclosamide (NSM) | | | | | | | | | | | | | | | | | | | | |

| **Table S3.** Demographic characteristics for all 41,399 residents who provided stool samples after the first round of niclosamide mass drug administration by those who were positive for taeniasis and those with a 30-day follow-up stool sample | | | | | | | | | | | | | | |
| --- | --- | --- | --- | --- | --- | --- | --- | --- | --- | --- | --- | --- | --- | --- |
|  | **Provided Stool Sample* N = 41,399** | |  | **Negative for Taeniasis N = 41,188** | |  | **Positive for Taeniasis N = 211** | |  | **Follow-up Stool Sample  N = 188** | |  | **No Follow-up Stool Sample  N = 23** | |
|  | n | % |  | n | % |  | n | % |  | n | % |  | n | % |
| **Age (Years),** mean (SD) | 30∙0 | 19∙7 |  | 30.0 | 19∙8 |  | 32∙0 | 17∙2 |  | 32∙2 | 17∙5 |  | 29.9 | 15∙5 |
| **Sex** |  |  |  |  |  |  |  |  |  |  |  |  |  |  |
| Male | 19,528 | 47∙2% |  | 19,431 | 47∙2% |  | 97 | 46∙0% |  | 85 | 45∙2% |  | 12 | 52∙2% |
| Female | 21,871 | 52∙8% |  | 21,757 | 52∙8% |  | 114 | 54∙0% |  | 103 | 54∙8% |  | 11 | 47∙8% |
| **Environment** |  |  |  |  |  |  |  |  |  |  |  |  |  |  |
| Urban | 21,232 | 51∙3% |  | 21,146 | 51∙3% |  | 86 | 40∙8% |  | 81 | 43∙1% |  | 5 | 21∙7% |
| Rural | 20,167 | 48∙7% |  | 20,042 | 48∙7% |  | 125 | 59∙2% |  | 107 | 56∙9% |  | 18 | 78∙3% |
| **Water source** |  |  |  |  |  |  |  |  |  |  |  |  |  |  |
| Public network | 25,743 | 62∙2% |  | 25,618 | 62∙2% |  | 125 | 59∙2% |  | 112 | 59∙6% |  | 13 | 56∙5% |
| No public network | 15,656 | 37∙8% |  | 15,570 | 37∙8% |  | 86 | 40∙8% |  | 76 | 40∙4% |  | 10 | 43∙5% |
| **Household sanitation** |  |  |  |  |  |  |  |  |  |  |  |  |  |  |
| Bathroom | 15,371 | 37∙1% |  | 15,306 | 37∙2% |  | 65 | 30∙8% |  | 57 | 30∙3% |  | 8 | 34∙8% |
| Latrine | 15,811 | 38∙2% |  | 15,714 | 38∙2% |  | 97 | 46∙0% |  | 88 | 46∙8% |  | 9 | 39∙1% |
| Neither | 10,217 | 24∙7% |  | 10,168 | 24∙7% |  | 49 | 23∙2% |  | 43 | 22∙9% |  | 6 | 26∙1% |
| **Electricity** |  |  |  |  |  |  |  |  |  |  |  |  |  |  |
| Public service | 35,414 | 85∙5% |  | 35,246 | 85∙6% |  | 168 | 79∙6% |  | 150 | 79∙8% |  | 18 | 78∙3% |
| No public service | 5,985 | 14∙5% |  | 5,942 | 14∙4% |  | 43 | 20∙4% |  | 38 | 20∙2% |  | 5 | 21∙7% |
| **Livestock** |  |  |  |  |  |  |  |  |  |  |  |  |  |  |
| Raises animals | 28,062 | 67∙8% |  | 27,905 | 67∙8% |  | 157 | 74∙4% |  | 140 | 74∙5% |  | 17 | 73∙9% |
| Does not raise animals | 13,337 | 32∙2% |  | 13,283 | 32∙2% |  | 54 | 25∙6% |  | 48 | 25∙5% |  | 6 | 26∙1% |
| **Antigen levels (PP)*** |  |  |  |  |  |  |  |  |  |  |  |  |  |  |
| Mean (SD) | 3∙7 | 5∙1 |  | 3∙5 | 1∙7 |  | 49∙1 | 50∙7 |  | 50∙6 | 51∙5 |  | 36∙4 | 42∙3 |
| Median (min, max) | 3∙1 | [0∙4, 251∙0] |  | 3∙1 | [0∙4, 20∙0] |  | 30∙0 | [1∙6, 251∙0] |  | 30∙0 | [1∙6, 251∙0] |  | 29∙9 | [2∙1, 210∙0] |
| Abbreviations: standard deviation (SD), niclosamide (NSM), percentage of positivity (PP)  *Based on stool sample collected 24 hours following NSM treatment | | | | | | | | | | | | | | |

# **Sensitivity Analysis Methods & Results**

## **Imputation Methods**

Multiple Imputation by Chained Equations (MICE) package in R was used to address missing data related to the three outcomes: taeniasis prevalence, niclosamide (NSM) effectiveness, and any adverse events following NSM. To construct each imputation model, independent variables that were associated with missing data or the outcome were selected (Table S5). For the imputation procedure, we set a seed value to ensure reproducibility and established a maximum of 20 iterations to allow for convergence. Predictive mean matching served as the imputation method for all continuous variables, while logistic regression (logreg) was employed for binary variables. Subsequently, we generated five sets of imputed datasets to capture uncertainty in the imputation process. The distributions of imputed values were compared to those in the analytic sample with no missing data to assess feasibility of output. Subsequently, the mean values for each outcome were computed across the five imputed datasets and reported.

**Imputation Results**

## **Table S4.** List of independent variables associated with missing data or study outcomes (taeniasis prevalence, niclosamide effectiveness, and any adverse events) used in imputation models.

| **Outcome** | **Variables associated with missing data** | **Variables associated with the outcome** |
| --- | --- | --- |
| Taeniasis prevalence | Younger mean age, male, and did not raise livestock | Living in a rural community, using a latrine, no public electricity, raises livestock |
| NSM effectiveness | Living in a rural community and being male | Lower coproantigen levels, younger age |
| Any adverse event(s) | Younger mean age, being male, and not raising livestock | Older mean age, being female, living in a rural community, having public water and electricity access, uses a latrine or no bathroom |

**Abbreviations:** niclosamide (NSM)

**Table S5.** Original and imputed outcome data accounting for missing data based on availability of stool samples and response to the any adverse event questionnaire.

|  | **Study Outcomes** | | |
| --- | --- | --- | --- |
|  | **Taeniasis Prevalence** | **NSM Effectiveness** | **Any Adverse Event(s)** |
| **Original Data** | 51∙0% | 75∙0% | 1∙51% |
| **Imputed Data**, Average | 50∙6% | 75∙3% | 1∙50% |
| Dataset 1 | 49∙1% | 76∙8% | 1∙48% |
| Dataset 2 | 52∙2% | 73∙9% | 1∙51% |
| Dataset 3 | 50∙1% | 75∙4% | 1∙50% |
| Dataset 4 | 52∙0% | 74∙9% | 1∙50% |
| Dataset 5 | 49∙9% | 75∙4% | 1∙52% |

**Abbreviations:** niclosamide (NSM)

## **Scenario Methods**

We conducted an analysis to estimate the effectiveness of niclosamide (NSM) treatment in both a best-case and worst-case scenario, focusing on the subset of 211 participants who tested positive for taeniasis based on their initial stool sample collected 24 hours after the first round of NSM administration. In the worst-case scenario, we assumed that the 23 participants who were missing a second stool sample at the 30-day follow-up remained infected. Conversely, in the best-case scenario, we assumed that all 23 participants had successfully cleared the infection by the 30-day follow-up. Additionally, we assessed scenarios that ranged in between where we assumed that 20%, 40%, 60%, and 80% of participants with missing the 30-day stool sample had cleared their infection.

## **Scenario Results**

**Table S6.** Original and missing data scenarios for estimated niclosamide (NSM) effectiveness.

| **Scenarios** | **Cured/Infected** | **NSM Effectiveness (95% CI)** |
| --- | --- | --- |
| **Original Data** | 141/188 | 75∙0% (68∙4%, 80∙6%) |
| **Scenarios** |  |  |
| 0% cleared | 141/211 | 66∙8% (60∙2%, 72∙8%) |
| 20% cleared | 146/211 | 69∙2% (62∙7%, 75∙0%) |
| 40% cleared | 150/211 | 71∙1% (64∙6%, 76∙8%) |
| 60% cleared | 155/211 | 73∙5% (67∙1%, 79∙0%) |
| 80% cleared | 159/211 | 75∙4% (69∙1%, 80∙7%) |
| 100% cleared | 164/211 | 77∙7% (71∙6%, 82∙8%) |

Scenarios applied to the 23 participants who were missing a second stool sample at 30-day follow-up and then added to the known number of participants who cleared their infection based on their follow-up test.
**Abbreviations:** niclosamide (NSM)

# **Any Adverse Event Stratified by Round**

**Table S7.** Participant characteristics according to the occurrence of any adverse event during niclosamide mass treatment implementation overall and stratified by treatment round, among 65,551 residents living in Tumbes, Peru between 2009-2010.

|  | **Overall**  **N = 65,551** | |  | **Round 1**  **N = 47,090** | |  | | **Round 2**  **N = 52,901** | | |  | | **Round 3**  **N = 54,224** | | |
| --- | --- | --- | --- | --- | --- | --- | --- | --- | --- | --- | --- | --- | --- | --- | --- |
|  | **Any AE** | **No AE** |  | **Any AE** | **No AE** |  | **Any AE** | | **No AE** |  | | **Any AE** | | **No AE** |  |
|  | **N = 988** | **N = 64,563** |  | **N = 130** | **N = 46,960** |  | **N = 479** | | **N = 52,422** |  | | **N = 398** | | **N = 53,826** |  |
|  | **n (%)** | **n (%)** |  | **n (%)** | **n (%)** |  | **n (%)** | | **n (%)** |  | | **n (%)** | | **n (%)** |  |
| **Participant Age (Years), mean (SD)** | 34∙0 (18∙70) | 29∙7 (19∙60) |  | 34.5 (19∙9) | 29∙2 (19∙5) |  | 34∙1 (18∙2) | | 29∙5 (19∙8) |  | | 34∙1 (18∙6) | | 29∙5 (19∙7) |  |
| **Age Categories (Years)** |  |  |  |  |  |  |  | |  |  | |  | |  |  |
| < 20 | 244 (24∙7%) | 24,454 (37∙9%) |  | 35 (26∙9%) | 18,879 (40∙2%) |  | 116 (24∙2%) | | 20,784 (39∙6%) |  | | 94 (23∙6%) | | 21,030 (39∙1%) |  |
| 20-39 | 386 (39∙1%) | 21,206 (32∙8%) |  | 41 (31∙5%) | 14,338 (30∙5%) |  | 189 (39∙5%) | | 15,994 (30∙5%) |  | | 163 (41∙0%) | | 16,893 (31∙4%) |  |
| 40-59 | 259 (26∙2%) | 13,216 (20∙5%) |  | 41 (31∙5%) | 9,865 (21∙0%) |  | 125 (26∙1%) | | 11,040 (21∙1%) |  | | 103 (25∙9%) | | 11,242 (20∙9%) |  |
| 60 + | 99 (10∙0%) | 5,687 (8∙8%) |  | 13 (10∙0%) | 3,878 (8∙3%) |  | 49 (10∙2%) | | 4,604 (8∙8%) |  | | 38 (9∙5%) | | 4,661 (8∙7%) |  |
| **Participant Sex** |  |  |  |  |  |  |  | |  |  | |  | |  |  |
| Male | 296 (30∙0%) | 33,090 (51∙3%) |  | 42 (32∙3%) | 22,888 (48∙7%) |  | 137 (28∙6%) | | 26,630 (50∙8%) |  | | 117 (29∙4%) | | 27,553 (51∙2%) |  |
| Female | 692 (70∙0%) | 31,473 (48∙7%) |  | 88 (67∙7%) | 24,072 (51∙3%) |  | 342 (71∙4%) | | 25,792 (49∙2%) |  | | 281 (70∙6%) | | 26,273 (48∙8%) |  |
| **Household Location** |  |  |  |  |  |  |  | |  |  | |  | |  |  |
| Rural | 561 (56∙8%) | 32,038 (49∙6%) |  | 59 (45∙4%) | 23,250 (49∙5%) |  | 304 (63∙5%) | | 26,203 (50∙0%) |  | | 212 (53∙3%) | | 27,349 (50∙8%) |  |
| Peri-urban | 427 (43∙2%) | 32,525 (50∙4%) |  | 71 (54∙6%) | 23,710 (50∙5%) |  | 175 (36∙5%) | | 26,219 (50∙0%) |  | | 186 (46∙7%) | | 26,477 (49∙2%) |  |
| **Number of Household Members, mean (SD)** | 4∙18 (1∙74) | 4∙24 (1∙66) |  | 4.25 (1∙58) | 4.27 (1∙61) |  | 4∙10 (1∙80) | | 4∙26 (1∙62) |  | | 4∙24 (1∙69) | | 4∙24 (1∙66) |  |
| **Household Access to Public Water Source** |  |  |  |  |  |  |  | |  |  | |  | |  |  |
| Yes | 436 (44∙1%) | 24,509 (38∙0%) |  | 42 (32∙3%) | 17,827 (38∙0%) |  | 214 (44∙7%) | | 19,981 (38∙1%) |  | | 188 (47∙2%) | | 20,635 (38∙3%) |  |
| No | 552 (55∙9%) | 40,054 (62∙0%) |  | 88 (67∙7%) | 29,133 (62∙0%) |  | 265 (55∙3%) | | 32,441 (61∙9%) |  | | 210 (52∙8%) | | 33,191 (61∙7%) |  |
| **Household Access to Public Electricity** |  |  |  |  |  |  |  | |  |  | |  | |  |  |
| Yes | 188 (19∙0%) | 9,485 (14∙7%) |  | 25 (19∙2%) | 6,685 (14∙2%) |  | 92 (19∙2%) | | 7,586 (14∙5%) |  | | 73 (18∙3%) | | 7,967 (14∙8%) |  |
| No | 800 (81∙0%) | 55,078 (85∙3%) |  | 105 (80∙8%) | 40,275 (85∙8%) |  | 387 (80∙8%) | | 44,836 (85∙5%) |  | | 325 (81∙7%) | | 45,859 (85∙2%) |  |
| **Household Sanitation** |  |  |  |  |  |  |  | |  |  | |  | |  |  |
| Bathroom | 277 (28∙0%) | 23,946 (37∙1%) |  | 42 (32∙3%) | 17,337 (36∙9%) |  | 111 (23∙2%) | | 19,387 (37∙0%) |  | | 127 (31∙9%) | | 19,628 (36∙5%) |  |
| Latrine | 416 (42∙1%) | 24,318 (37∙7%) |  | 51 (39∙2%) | 17,926 (38∙2%) |  | 217 (45∙3%) | | 19,904 (38∙0%) |  | | 156 (39∙2%) | | 20,545 (38∙2%) |  |
| None | 295 (29∙9%) | 16,299 (25∙2%) |  | 37 (28∙5%) | 11,697 (24∙9%) |  | 151 (31∙5%) | | 13,131 (25∙0%) |  | | 115 (28∙9%) | | 13,653 (25∙4%) |  |
| **Household Raises Livestock** |  |  |  |  |  |  |  | |  |  | |  | |  |  |
| Yes | 653 (66∙1%) | 42,929 (66∙5%) |  | 83 (63∙8%) | 31,907 (67∙9%) |  | 319 (66∙6%) | | 35,075 (66∙9%) |  | | 261 (65∙6%) | | 36,273 (67∙4%) |  |
| No | 335 (33∙9%) | 21,634 (33∙5%) |  | 47 (36∙2%) | 15,053 (32∙1%) |  | 160 (33∙4%) | | 17,347 (33∙1%) |  | | 137 (34∙4%) | | 17,553 (32∙6%) |  |

**Abbreviations:** adverse event (AE), standard deviation (SD)

**Table S8.** Severity and types of adverse events reported among participants who responded to the adverse event questionnaire (AEQ) over multiple rounds of niclosamide (NSM) mass treatment and stratified by treatment round in Tumbes, Peru, 2009-2010.

|  | **Overall** | **Round 1** | **Round 2** | **Round 3** |
| --- | --- | --- | --- | --- |
|  | **N=988** | **N=130** | **N=479** | **N=398** |
| **Adverse Event** | **n (%)** | **n (%)** | **n (%)** | **n (%)** |
| **Severity of adverse event** |  |  |  |  |
| Severe | 0 (0∙0%) | 0 (0∙0%) | 0 (0∙0%) | 0 (0∙0%) |
| Moderate | 8 (0∙8%) | 6 (4∙6%) | 0 (0∙0%) | 2 (0∙5%) |
| Mild | 980 (99∙2%) | 124 (95∙4%) | 479 (100%) | 396 (99∙5%) |
| **Type of adverse event** |  |  |  |  |
| Abdominal discomfort | 557 (56∙4%) | 58 (44∙6%) | 275 (57∙4%) | 233 (58∙5%) |
| Headache | 243 (24∙6%) | 30 (23∙1%) | 127 (26∙5%) | 87 (21∙9%) |
| Tongue numbness | 141 (14∙3%) | 16 (12∙3%) | 66 (13∙8%) | 62 (15∙6%) |
| Diarrhea | 136 (13∙8%) | 24 (18∙5%) | 56 (11∙7%) | 56 (14∙1%) |
| Nausea | 98 (9∙9%) | 16 (12∙3%) | 50 (10∙4%) | 32 (8∙0%) |
| Watery stool | 80 (8∙1%) | 10 (7∙7%) | 41 (8∙6%) | 29 (7∙3%) |
| Vomiting | 53 (5∙4%) | 14 (10∙8%) | 21 (4∙4%) | 18 (4∙5%) |
| Dizziness | 50 (5∙1%) | 11 (8∙5%) | 21 (4∙4%) | 18 (4∙5%) |
| Rash | 33 (3∙3%) | 16 (12∙3%) | 12 (2∙5%) | 6 (1∙5%) |
| Other types of pain | 30 (3∙0%) | 14 (10∙8%) | 11 (2∙3%) | 5 (1∙3%) |
| Discomfort | 29 (2∙9%) | 7 (5∙4%) | 15 (3∙1%) | 7 (1∙8%) |
| Pruritus | 16 (1∙6%) | 7 (5∙4%) | 6 (1∙3%) | 3 (0∙8%) |
| Constipation | 12 (1∙2%) | 6 (4∙6%) | 5 (1∙0%) | 1 (0∙3%) |
| Abdominal distension | 9 (0∙9%) | 1 (0∙8%) | 6 (1∙3%) | 2 (0∙5%) |
| Epigastric burning | 6 (0∙6%) | 0 (0∙0%) | 4 (0∙8%) | 2 (0∙5%) |
| Bitter Taste | 4 (0∙4%) | 2 (1∙5%) | 2 (0∙4%) | 0 (0∙0%) |
| Fever | 4 (0∙4%) | 1 (0∙8%) | 1 (0∙2%) | 2 (0∙5%) |
| Other | 104 (10∙5%) | 2 (1∙5%) | 35 (7∙5%) | 67 (16∙8%) |
| **Number of total adverse event types reported** |  |  |  |  |
| Mean (SD) | 1∙64 (0∙78) | 1.81 (0.87) | 1∙57 (0∙73) | 1∙58 (0∙67) |
| Median (Min, Max) | 1∙00 (1∙00, 6∙00) | 2∙00 [1∙00, 5∙00] | 1∙00 [1∙00, 5∙00] | 1∙00 [1∙00, 4∙00] |
| **Number of total adverse event types reported** |  |  |  |  |
| 1 | 502 (51∙2%) | 59 (45∙4%) | 263 (54∙9%) | 201 (50∙5%) |
| 2 | 372 (38∙0%) | 41 (31∙5%) | 166 (34∙7%) | 168 (42∙2%) |
| 3+ | 114 (11∙5%) | 30 (23∙1%) | 50 (10∙4%) | 29 (7∙3%) |

# **Unrelated adverse events**

**Table S9.** Types of adverse events unrelated to niclosamide (NSM) overall and stratified by severity reported by 100 participants following mass treatment with NSM, Tumbes, Peru, 2009-2010.

|  | **All Unrelated AE** | **Mild** | **Moderate** | **Severe** |
| --- | --- | --- | --- | --- |
| **Type of adverse event** | **N = 100 n (%)** | **N = 96**  **n (%)** | **N = 3**  **n (%)** | **N = 1**  **n (%)** |
| Abdominal discomfort | 46 (46∙0%) | 44 (45∙8%) | 2 (66∙7%) | 0 (0%) |
| Fever | 28 (28∙0%) | 24 (25∙0%) | 3 (100%) | 1 (100%) |
| Diarrhea | 23 (23∙0%) | 23 (24∙0%) | 0 (0%) | 0 (0%) |
| Headache | 21 (21∙0%) | 20 (20∙8%) | 1 (33∙3%) | 0 (0%) |
| Vomiting | 20 (20∙0%) | 19 (19∙8%) | 1 (33∙3%) | 0 (0%) |
| Other types of pain | 19 (19∙0%) | 19 (19∙8%) | 0 (0%) | 0 (0%) |
| Discomfort | 17 (17∙0%) | 16 (16∙7%) | 1 (33∙3%) | 0 (0%) |
| Nausea | 8 (8∙0%) | 8 (8∙3%) | 0 (0%) | 0 (0%) |
| Dizziness | 8 (8∙0%) | 8 (8∙3%) | 0 (0%) | 0 (0%) |
| Sore throat | 6 (6∙0%) | 6 (6∙3%) | 0 (0%) | 0 (0%) |
| Other | 6 (6∙0%) | 6 (6∙3%) | 0 (0%) | 0 (0%) |
| Watery stool | 5 (5∙0%) | 5 (5∙2%) | 0 (0%) | 0 (0%) |
| Epigastric burning | 4 (4∙0%) | 4 (4∙2%) | 0 (0%) | 0 (0%) |
| Joint pain | 3 (3∙0%) | 3 (3∙1%) | 0 (0%) | 0 (0%) |
| Dyspnea | 2 (2∙0%) | 2 (2∙1%) | 0 (0%) | 0 (0%) |
| Constipation | 1 (1∙0%) | 1 (1∙0%) | 0 (0%) | 0 (0%) |
| Milia | 1 (1∙0%) | 1 (1∙0%) | 0 (0%) | 0 (0%) |
| Pruritus | 1 (1∙0%) | 1 (1∙0%) | 0 (0%) | 0 (0%) |
| Rash | 1 (1∙0%) | 1 (1∙0%) | 0 (0%) | 0 (0%) |

# **Recurrent Adverse Events during NSM Treatment Rounds**

**Table S10.** Participant characteristics among those who reported adverse events (AE) following multiple NSM treatment rounds (n = 18)

|  | **Recurrent AE** |
| --- | --- |
|  | **N = 18** |
|  | **n (%)** |
| **Round participation** |  |
| All rounds | 16 (88.9%) |
| Rounds 1 and 2 | 1 (5.6%) |
| Rounds 2 and 3 | 1 (5.6%) |
| **Participant Age (Years)** |  |
| Mean (SD) | 41.4 (13.7) |
| Median [Min, Max] | 46.5 [18.0, 67.0] |
| **Participant Sex** |  |
| Male | 0 (0%) |
| Female | 18 (100%) |
| **Household Location** |  |
| Rural | 13 (72.2%) |
| Peri-urban | 5 (27.8%) |
| **Number of Household Members, mean (SD)** | 4.06 (0.938) |
| **Household Access to Public Water Source** |  |
| Yes | 8 (44.4%) |
| No | 10 (55.6%) |
| **Household Access to Public Electricity** |  |
| Yes | 2 (11.1%) |
| No | 16 (88.9%) |
| **Household Sanitation** |  |
| Bathroom | 3 (16.7%) |
| Latrine | 7 (38.9%) |
| None | 8 (44.4%) |
| **Household Raises Livestock** |  |
| Yes | 9 (50.0%) |
| No | 9 (50.0%) |

**Abbreviations:** adverse event (AE), standard deviation (SD)

**Table S11.** Types of adverse events (AE) reported among participants who reported AEs following multiple NSM treatment rounds (n = 18)

|  | **Recurrent AE** | |
| --- | --- | --- |
|  | **N = 18** | |
|  | **Any AE Reported Overall** | **Same AE Reported in Multiple Rounds** |
| **Type of adverse event** | **N (%)** | **N (%)** |
| Any abdominal discomfort | 12 (66.7%) | 8 (44.4%) |
| Headache | 11 (61.1%) | 1 (5.6%) |
| Tongue numbness | 6 (33.3%) | 3 (16.7%) |
| Diarrhea | 3 (16.7%) | 0 (0.0%) |
| Nausea | 2 (11.1%) | 0 (0.0%) |
| Watery stool | 2 (11.1%) | 0 (0.0%) |
| Dizziness | 2 (11.1%) | 0 (0.0%) |
| Abdominal distension | 2 (11.1%) | 0 (0.0%) |
| Rash | 1 (5.6%) | 1 (5.6%) |
| Vomiting | 1 (5.6%) | 0 (0.0%) |
| Other types of pain | 1 (5.6%) | 0 (0.0%) |
| Discomfort | 1 (5.6%) | 0 (0.0%) |
| Epigastric burning | 1 (5.6%) | 0 (0.0%) |
| Other | 1 (5.6%) | 0 (0.0%) |

All participants reported adverse events of mild intensity only. **Abbreviations:** adverse event (AE), standard deviation (SD)

# **Subgroup Analysis**

## **NSM Effectiveness**

**Table S12.** Niclosamide (NSM) effectiveness by age category among participants positive for taeniasis and with a stool sample 30 days following the first round of the niclosamide mass drug administration in Tumbes, Peru, June-August 2009.

| **Age Category** | **N** | **n (%)*** |
| --- | --- | --- |
| 2-6 years | 2 | 2 (100%) |
| 7-12 years | 20 | 18 (90∙0%) |
| 13-19 years | 35 | 25 (71∙4%) |
| 20+ years | 131 | 96 (73∙3%) |
| All ages | 188 | 141 (75∙0%) |

*Row percentages among participants in each age group

## **Any Adverse Event**

**Table S13.** Any adverse events by age category among participants who responded to the adverse event questionnaire (AEQ) following multiple rounds of niclosamide (NSM) mass treatment in Tumbes, Peru, 2009-2010.

| **Age Category** | **N** | **n (%)*** |
| --- | --- | --- |
| 2-6 years | 6,549 | 57 (0∙9%) |
| 7-12 years | 8,559 | 98 (1∙1%) |
| 13-19 years | 9,590 | 89 (0∙9%) |
| 20+ years | 40,853 | 744 (1∙8%) |
| All ages | 65,551 | 988 (1∙5%) |

*Row percentages among participants in each age group

**Table S14.** Any adverse events by sex among participants who responded to the adverse event questionnaire (AEQ) following multiple rounds of niclosamide (NSM) mass treatment in Tumbes, Peru, 2009-2010.

|  | **Female** | **Male** |
| --- | --- | --- |
|  | **N=692** | **N=296** |
| **Adverse Event** | **n (%)** | **n (%)** |
| **Severity of adverse event** |  |  |
| Severe |  |  |
| Moderate | 0 (0∙0%) | 0 (0∙0%) |
| Mild | 6 (0∙9%) | 2 (0∙7%) |
| **Type of adverse event** | 686 (99∙1%) | 294 (99∙3%) |
| Abdominal discomfort |  |  |
| Headache | 386 (55∙8%) | 171 (57∙8%) |
| Tongue numbness | 188 (27∙2%) | 55 (18∙6%) |
| Diarrhea | 115 (16∙6%) | 26 (8∙8%) |
| Nausea | 82 (11∙8%) | 54 (18∙2%) |
| Watery stool | 73 (10∙5%) | 25 (8∙4%) |
| Vomiting | 53 (7∙7%) | 27 (9∙1%) |
| Dizziness | 39 (5∙6%) | 14 (4∙7%) |
| Rash | 6 (0∙9%) | 6 (2∙0%) |
| Other types of pain | 37 (5∙3%) | 13 (4∙4%) |
| Discomfort | 16 (2∙3%) | 17 (5∙7%) |
| Pruritus | 23 (3∙3%) | 7 (2∙4%) |
| Constipation | 19 (2∙7%) | 10 (3∙4%) |
| Abdominal distension | 12 (1∙7%) | 4 (1∙4%) |
| Epigastric burning | 6 (0∙9%) | 3 (1∙0%) |
| Bitter Taste | 3 (0∙4%) | 3 (1∙0%) |
| Fever | 3 (0∙4%) | 1 (0∙3%) |
| Other | 3 (0∙4%) | 1 (0∙3%) |
| **All adverse events reported, count** | 71 (10∙1%) | 33 (11∙1%) |
| Mean (SD) |  |  |
| Median (Min, Max) | 1∙66 (0∙82) | 1∙59 (0∙68) |
| **All adverse event reported, categorical** | 1∙00 (1∙00, 6∙00) | 1∙00 (1∙00, 4∙00) |
| 1 |  |  |
| 2 | 352 (50∙9%) | 150 (50∙7%) |
| 3+ | 250 (36∙1%) | 122 (41∙2%) |
| **Any recurrent adverse event(s)** | 90 (13∙0%) | 24 (8∙1%) |
| None – AE occurred in one round only |  |  |
| AE occurred in two rounds | 674 (97∙4%) | 296 (100%) |
| AE occurred in three rounds | 17 (2∙5%) | 0 (0∙0%) |
