# Supplementary material for: Mass chemotherapy with niclosamide for the control of Taenia solium: population-based safety profile and treatment effectiveness
Source: Lancet Reg Health Am. 2024 Aug 30;38:100876. doi: 10.1016/j.lana.2024.100876 (PMC11402444; doi:10.1016/j.lana.2024.100876)
Supplement: Members-table [file mmc3.docx]

| Manuela R. | Verastegui |
| --- | --- |
| Javier A. | Bustos |
| Mirko | Zimic |
| Isidro | Gonzales |
| Herbert | Saavedra |
| Sofia S. | Sanchez |
| Manuel | Martinez |
| Yesenia | Castillo |
| Luz | Toribio |
| Gianfranco | Arroyo |
| Miguel A. | Orrego |
| Nancy | Chile |
| Holger | Mayta |
| Monica | Pajuelo |
| Saul | Santivañez |
| Eloy | Gonzalez-Gustavson |
| Luis | Gomez-Puerta |
| Cesar M. | Gavidia |
| Ana | Vargas-Calla |
| Maria T. | Lopez |
| Theodore E. | Nash |
| Sukwan | Handali |
| John | Noh |
| Jon | Friedland |

**NAMES OF STUDY GROUP MEMBERS**
